# Respiratory Variability in Disorders of Consciousness: Relationship and Clinical Applications

**DOI:** 10.1002/cns.70812

**Published:** 2026-05-12

**Authors:** Yongli Wu, Huaping Pan, Hui Feng, Juanjuan Fu, Yuqing Han

**Affiliations:** ^1^ Department of Rehabilitation Medicine The First People's Hospital of Foshan (Foshan Hospital Affiliated to Southern University of Science and Technology), School of Medicine, Southern University of Science and Technology Foshan China; ^2^ Rehabilitation Medicine Center Suzhou Kowloon Hospital Shanghai Jiao Tong University School of Medicine Suzhou China; ^3^ Rehabilitation Medicine Center Jiangning Hospital Affiliated to Nanjing Medical University Nanjing China

**Keywords:** bootstrap resampling, CRS‐R scoring, disorders of consciousness, generalized additive model, machine learning, Random Forest, respiratory variability

## Abstract

**Background:**

Respiratory variability (RV) reflects the dynamic modulation of breathing patterns by the central nervous system and may serve as a physiological marker of consciousness. However, its predictive value in disorders of consciousness (DOC) remains unclear.

**Objective:**

To investigate the relationship between RV and the level of consciousness, and to evaluate the potential of RV‐based analysis for predicting clinical outcomes in DOC patients.

**Methods:**

Patients with disorders of consciousness and healthy controls were assessed using inertial measurement unit (IMU) sensors to record triaxial acceleration signals. RV indicators were extracted from respiratory waveforms. A generalized additive model (GAM) was applied to adjust for confounding variables. Group differences were evaluated using Bootstrap resampling and the Mann–Whitney *U* test. Machine learning models—including random forest, elastic net, support vector machine, and partial least squares regression—were employed to predict Coma Recovery Scale–Revised (CRS‐R) scores and clinical outcomes.

**Results:**

Significant differences in multiple RV indicators were observed between patient and control groups (*p* < 0.05), indicating an association between RV and the pathophysiology of consciousness disorders. Among the tested models, the random forest algorithm achieved the best predictive performance for CRS‐R scores (mean squared error = 3.76, *R* ^2^ = 0.76) and for clinical outcomes (AUC = 0.74, sensitivity = 0.86), outperforming other models.

**Conclusions:**

Respiratory variability, particularly when analyzed via random forest modeling, shows strong potential for prognostic assessment and clinical decision support in disorders of consciousness. RV may represent a non‐invasive biomarker reflecting the neural control of respiration and consciousness state.

## Graphical abstract

Respiratory variability (RV) was analyzed in patients with consciousness disorders and healthy controls. Significant RV differences were found, with random forest models outperforming others in predicting clinical outcomes.

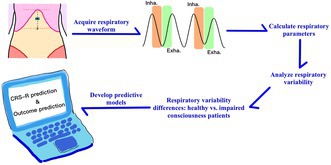

## 1 Introduction

DOC results from cardiac arrest, traumatic brain injury, intracranial hemorrhage, and other causes, leading to impairments or loss of wakefulness, attention, consciousness, and cognitive functions. Despite advances in intensive care technology that have improved the survival rates of patients with severe brain injuries, their quality of life remains poor, imposing significant burdens on individuals, families, and society [1, 2]. Thus, accurately assessing the level of consciousness and predicting clinical outcomes are urgent clinical needs.

Currently, the classification of DOC primarily relies on the observation of behavioral characteristics, such as assessments using the Coma Recovery Scale–Revised (CRS‐R). However, behavioral assessments are subjective and susceptible to observer bias, leading to uncertainty in results. While objective measures like functional magnetic resonance imaging (fMRI) and electroencephalography (EEG) can identify covert consciousness in patients without behavioral responses and suggest prognoses, these techniques are complex, costly, and challenging to implement in comatose or semi‐comatose patients [3, 4]. Therefore, developing an objective, convenient, and applicable method for assessing patients with DOC is particularly important.

In this context, the relationship between the autonomic nervous system (ANS) activity and DOC has gained increasing attention. Research indicates that most patients with brain trauma exhibit autonomic dysfunction, such as irregularities in heart rate, blood pressure, and respiratory regulation, which may be associated with cortical or subcortical damage. The central autonomic network (CAN) model describes the bidirectional interactions between the ANS and the central nervous system, involving areas like the anterior cingulate cortex, insula, amygdala, hypothalamus, and brainstem, highlighting that ANS function is an important indicator for assessing the prognosis of DOC [5, 6, 7].

Respiratory variability (RV) as a physiological indicator of ANS activity reflects the stability of an individual's physiological rhythms at rest. RV indicates the degree of fluctuation in respiratory parameters such as respiratory rate, tidal volume, inhalation time, and exhalation time over a certain period. Respiratory regulation is a dynamic balance network involving the control of the central nervous system, feedback from sensors, and the coordinated action of effectors [8]. The ANS influences the activity of the respiratory center and peripheral effectors through the regulation of sympathetic and parasympathetic nervous systems. Autonomic afferent fibers regulate the activity of respiratory‐related neuromuscular, airway, and pulmonary vascular functions, directly affecting the fluctuation of respiratory parameters [9].

In recent years, RV has been shown to be associated with various health conditions, such as cardiovascular diseases, sleep disorders, and mental stress [10, 11, 12]. However, the application and significance of RV in patients with DOC have not been fully studied. Considering that patients with DOC may have dysfunction in the autonomic nervous system and that the measurement of RV is convenient, real‐time, dynamic, and relatively stable, it has the potential to be an objective indicator for assessing the level of consciousness. Therefore, exploring the relationship between RV and consciousness levels has important clinical value.

Unlike heart rate variability, unified standards for RV analysis have not yet been established. Common methods include time‐domain analysis, frequency‐domain analysis, and nonlinear analysis. Time‐domain analysis is based on the time series of respiratory parameters, calculating statistics such as mean, standard deviation, coefficient of variation, root mean square of successive differences, etc. to reflect the overall level and distribution characteristics of RV [13, 14]. Mauo‐Ying et al. studied the application of RV in weaning from mechanical ventilation using key indicators from Poincaré plots of respiratory parameters [15]. Moreover, the triangular index, a short‐term fluctuation indicator of heart rate variability, has been widely applied in cardiac health assessments [16]. Therefore, this study quantifies RV using the standard deviation, coefficient of variation, root mean square of successive differences, triangular index, and key indicators from Poincaré plots of 300‐s respiratory parameters.

This study aims to explore the potential correlation between RV and consciousness levels by comparing RV between patients with DOC and the normal population. Our goal is to identify RV indicators that can be used to objectively assess consciousness levels, thus providing more accurate and convenient assessment tools for clinical use, and offering new insights for the prognostic assessment and rehabilitation decisions of patients with DOC.

## 2 Materials and Methods

### 2.1 Participants

This study recruited 46 patients with DOC due to ischemic–hypoxic or traumatic brain injury, who were undergoing treatment in the intensive care rehabilitation unit of Jiangning Hospital affiliated with Nanjing Medical University. Inclusion criteria included: (1) age between 18 and 75 years; (2) diagnosis of either vegetative state (VS) or minimally conscious state (MCS) according to criteria [17, 18]; (3) stable vital signs; (4) no hydrocephalus or severe brain atrophy, no major organ failure, and no use of sedative drugs in the past 7 days; (5) informed consent duly signed by the patients' families. Exclusion criteria included: (1) severe neurological or systemic disease; in a critical condition such as hemodynamically unstable or requiring mechanical ventilation due to respiratory failure; (2) occurrence of epileptic seizures during treatment; (3) use of drugs affecting cortical excitability other than levodopa and baclofen. Additionally, 44 healthy controls were recruited. Ultimately, data from 44 patients were included in the analysis (data from one patient with Biot's respiration and one patient who provided incorrect age were excluded).

This study was approved by the Ethics Committee of Jiangning Hospital affiliated with Nanjing Medical University (approval number: 2021–03‐024‐K01), and informed consent was obtained from all participants before their involvement in the study.

**FIGURE 1:**
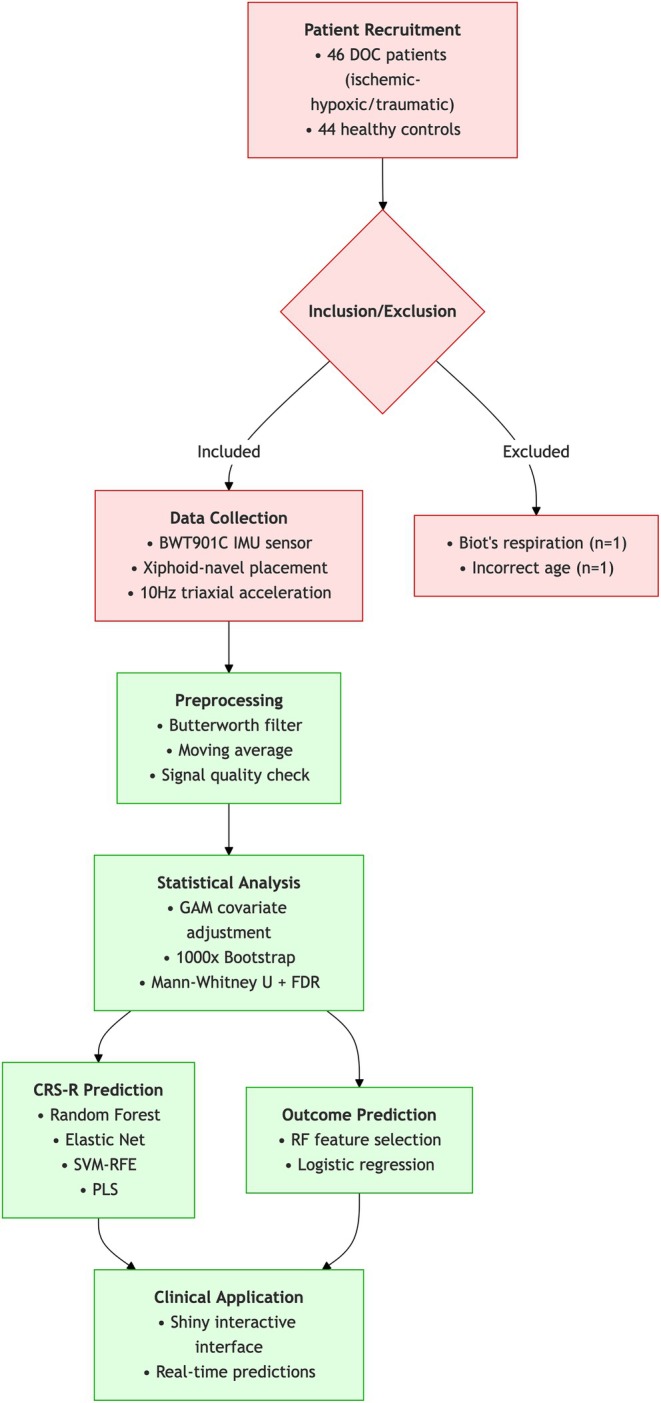
Experimental design for respiratory variability study in disorders of consciousness.

The complete analytical pipeline is visualized in Figure 1, showing participant flow from recruitment to final analysis.

### 2.2 Sensor Performance and Placement

**FIGURE 2:**
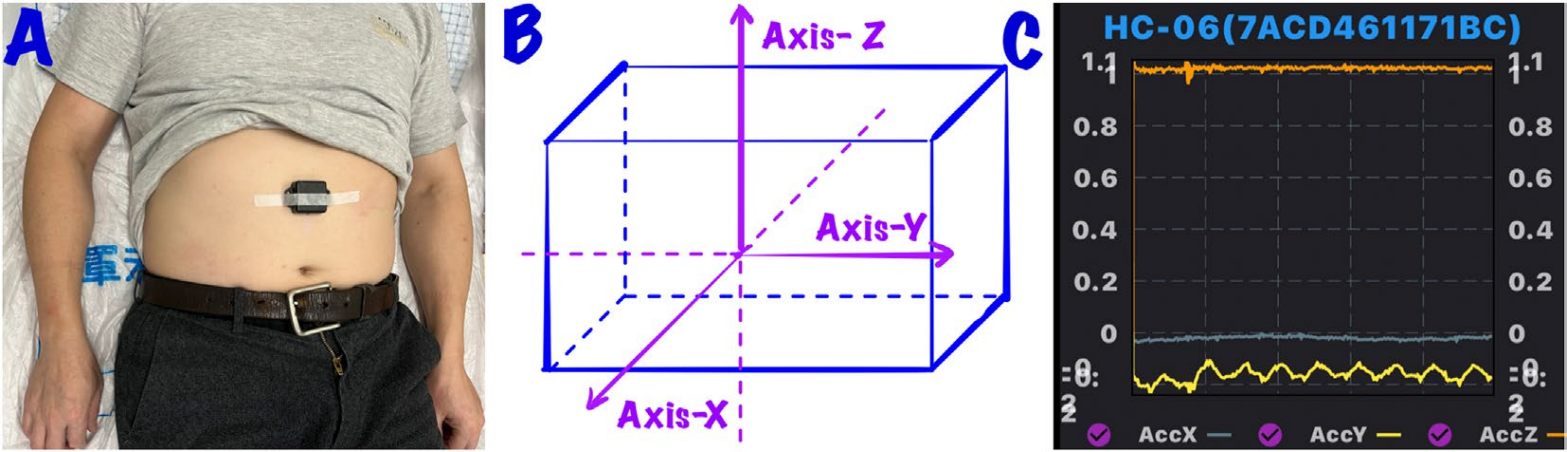
(A) The subject lying supine on a bed; (B) The axes of the sensor; (C) The interface of the WitMotion app.

In this study, we utilized the BWT901C sensor system equipped with an Inertial Measurement Unit (IMU), which transmits data in real‐time via Bluetooth. The sensor was positioned between the xiphoid process and the navel of the participants to collect triaxial acceleration data. (See Figure 2).

### 2.3 Signal Collection and Processing

**FIGURE 3:**
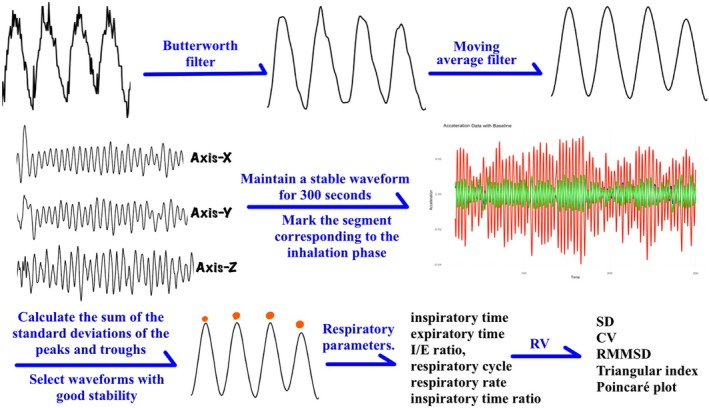
Diagram illustrating the collection of respiratory parameters and respiratory variability. CV, coefficient of variation; I/E ratio, inspiratory‐expiratory ratio; RMMSD, root mean square of successive differences; SD, standard deviation.

The data were collected at a sampling frequency of 10 Hz and initially processed in the R programming language for waveform plotting and filtering. Clear respiratory signals were extracted using Butterworth filtering and moving average techniques. Subsequently, a comprehensive analysis of the signals in both time and frequency domains was conducted to extract key respiratory parameters. (See Figure 3). Sensor measurement accuracy was validated in a separate cohort (see Supporting Information for validation details).

### 2.4 Experimental Methods

Under consistent environmental conditions, participants were positioned supine on a hard board while maintaining quiet and steady breathing. Sensors were secured between the xiphoid process and the navel using adhesive tape to collect data. This approach enabled the collection of respiratory parameters and indices of RV, along with basic confounding factors such as age, gender, body temperature, heart rate, and pulse pressure differences from both healthy individuals and patients with DOC. For the latter group, the level of consciousness was assessed using the CRS‐R, and the data were analyzed using statistical methods.

### 2.5 Data Processing and Statistical Analysis

**FIGURE 4:**
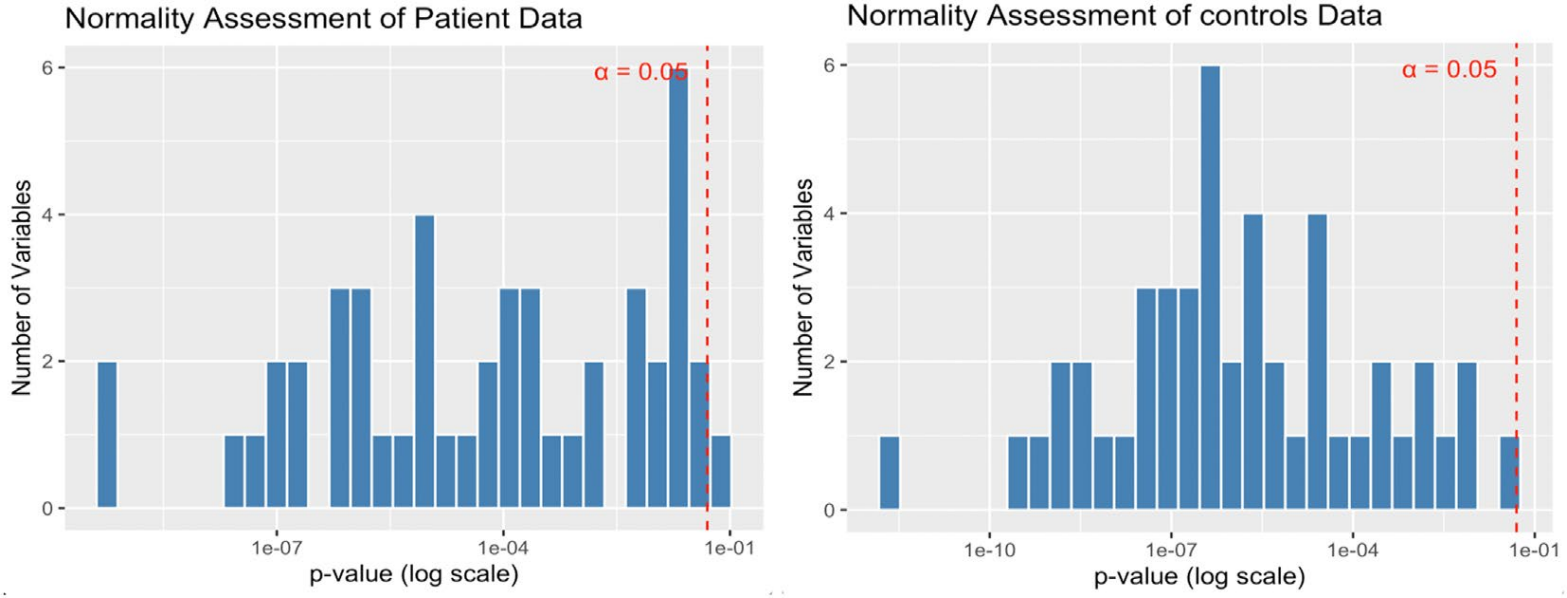
Shapiro–Wilk test between patients and controls group.

In this study, we initially conducted data cleaning by removing missing values and extracting the necessary indices of RV and confounding factors from the raw data. A GAM was utilized to control for confounding factors such as age, gender, body temperature, heart rate, and pulse pressure differences. Subsequently, the adjusted data for both the patient group and the control group were subjected to 1000 rounds of Bootstrap resampling to ensure the stability and precision of the statistics [19]. Given the non‐normal distribution of RV metrics (Shapiro–Wilk test, *p* < 0.05 for most indices) (See Figure 4), inter‐group differences were evaluated using the non‐parametric Mann–Whitney *U* test, and results were corrected for multiple comparison errors using the FDR method. Significant findings were visually presented in graphs and saved in an Excel file. Data cleaning involved a thorough check and removal of missing values to ensure the integrity of the data analyzed.

### 2.6 Constructing the CRS‐R Prediction Model

In this study, we employed four predictive models: Random Forest, Elastic Net Regression, Support Vector Machine Recursive Feature Elimination (SVM‐RFE), and Partial Least Squares Regression (PLS) to assess the scores of the CRS‐R. After hyperparameter tuning, cross‐validation, and selection of important features, each model was evaluated and compared using metrics such as Mean Squared Error (MSE), Root Mean Squared Error (RMSE), Mean Absolute Error (MAE), and Coefficient of Determination (*R* ^2^). The results were visualized using heatmaps and radar charts. Additionally, an interactive interface based on the Shiny framework was developed, allowing clinicians to input key features to predict CRS‐R scores. Data processing and analysis in this study were primarily conducted using the R language, involving packages such as mgcv, boot, ggplot2, openxlsx, mlr3, and randomForest.

### 2.7 Constructing the Outcome Prediction Model

This study assessed the clinical outcomes of patients with DOC using the CRS‐R. Patients were categorized as either in a vegetative state/unresponsive wakefulness syndrome (VS/UWS), characterized by eye‐opening without signs of consciousness, or in a minimally conscious state (MCS), displaying minimal signs of consciousness such as visual tracking or response to painful stimuli. Improved clinical outcomes were defined as a patient's improvement from VS/UWS to MCS or better, or further recovery within the MCS state. Unimproved outcomes included patients remaining in VS/UWS, no progression in MCS, or worsening conditions [20].

In this study, we utilized Elastic Net Regression and Random Forest models for feature selection and constructed logistic regression models for prediction. Data preprocessing included normalization and binary transformation, with feature selection determined through leave‐one‐out cross‐validation (LOOCV) and random search for optimal parameters. Model evaluation was performed using stratified sampling of training and validation sets, with Receiver Operating Characteristic (ROC) curves, Area Under the Curve (AUC), accuracy, sensitivity, specificity, and F1 scores as metrics. Additionally, we directly applied the Random Forest model for predictions and developed an interactive interface based on the Shiny framework, allowing real‐time prediction of patient clinical outcomes to support medical decision‐making.

## 3 Results

### 3.1 Baseline Characteristics Analysis

As demonstrated in Table 1, while not all baseline characteristics showed statistically significant differences between groups, clinically relevant variations were observed in gender distribution, age, hemodynamic parameters (blood pressure and pulse pressure), and body temperature. Given the established physiological relationships between these covariates and respiratory variability (RV) metrics [14], we employed generalized additive models (GAMs) to adjust for their potential confounding effects. This analytical approach ensured that the observed between‐group differences in RV parameters specifically reflected neural correlates of consciousness rather than systemic physiological variations.

**TABLE 1:** Baseline data of healthy individuals and patients with DOC.

	Sex (M/F)	Age (years)	SBP (mmHg)	DBP (mmHg)	PPD (mmHg)	*T* (°C)	HR (b/min)
Patients	13/44	55.8 ± 17.8	123.2 ± 17.5	76.8 ± 13.8	46.5 ± 13	36.6 ± 0.4	83.6 ± 16
Controls	22/44	45.8 ± 17	124.7 ± 14	79.3 ± 8	45.4 ± 9.8	36.4 ± 0.2	71.6 ± 11.7
*p*	0.231^a^	0.973^b^	0.007^b^	< 0.001^b^	0.011^b^	0.668^c^	0.114^b^

Abbreviations: DBP, diastolic blood pressure; HR, heart rate; PPD, pulse pressure difference; SBP, systolic blood pressure.

^a^ Fisher's exact test.

^b^ Mann–Whitney test.

^c^ *t*‐test.

### 3.2 RV Analysis

**FIGURE 5:**
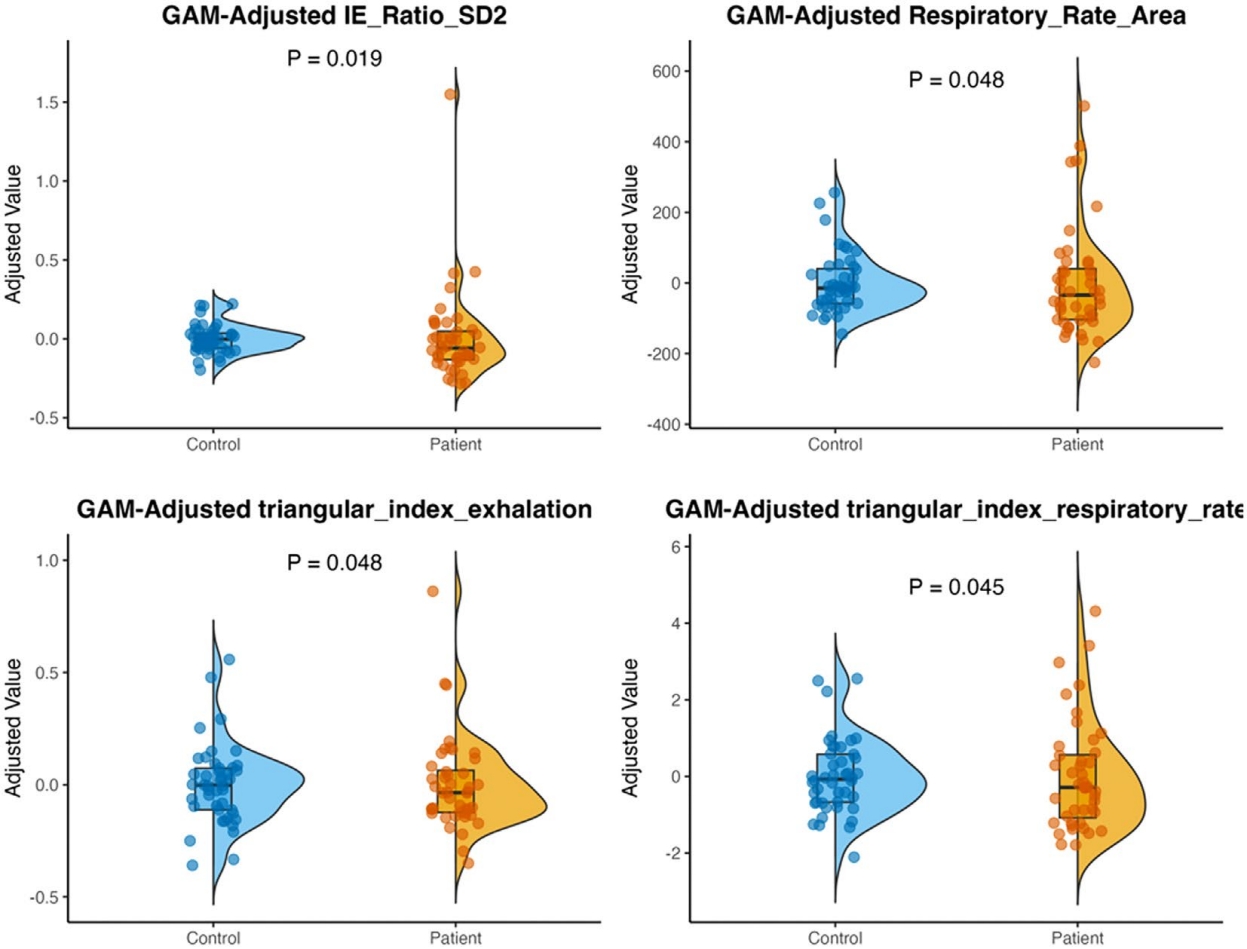
Group comparisons of GAM‐adjusted respiratory variability metrics (integrated visualization with violin plots, boxplots, and individual data points).

After adjusting for confounding factors such as age, gender, body temperature, heart rate, and pulse pressure difference, we conducted a Bootstrap resampling analysis to evaluate the differences in RV between the two groups. The results showed significant differences in multiple indices of RV between the patient group and the normal group (all *p*‐values < 0.05). Specifically, the inspiratory‐expiratory ratio SD2 was significantly higher in the DOC group, indicating increased complexity of respiratory rhythm. Conversely, the Respiratory Rate Area, as well as the triangular index of exhalation and respiratory rate, were significantly lower in the control group, reflecting reduced RV (see Figure 5). These results suggest that there are marked differences in RV features between patients with DOC and healthy individuals, likely related to disturbances in autonomic nervous function.

### 3.3 Construction of CRS‐R Prediction Model

**FIGURE 6:**
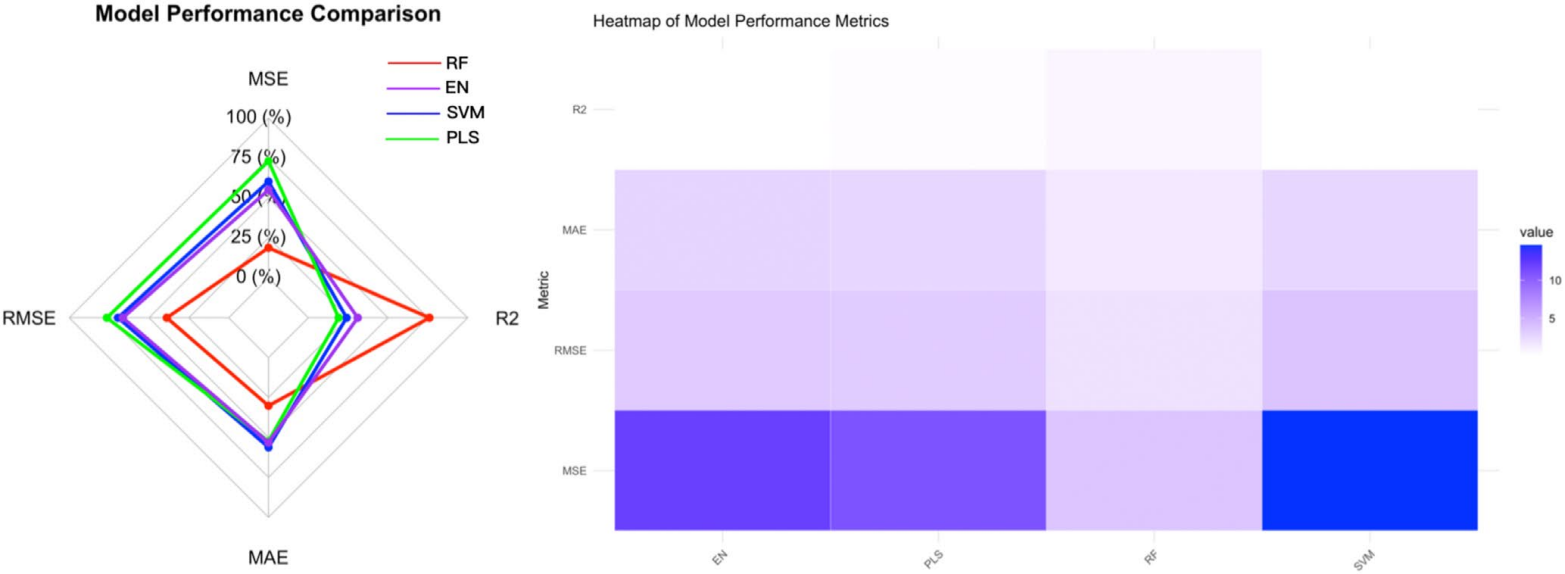
Comparison of predictive performance metrics for the four models. EN, Elastic Net; Error Metrics (MSE/RMSE/MAE): Lower values indicate better model performance; PLS, Partial Least Squares; *R* ^2^, Higher values indicate better fit; RF, Random Forest; SVM: Support Vector Machine.

The results showed that the Random Forest model performed the best, with an MSE of 3.76, RMSE of 1.94, MAE of 1.51, and an *R* ^2^ of 0.76, significantly outperforming the other models. The Support Vector Machine exhibited poorer predictive performance with an MSE of 14.63, RMSE of 3.82, MAE of 2.62, and an *R* ^2^ of only 0.19. The performance of Elastic Net and Partial Least Squares was intermediate between the two, displaying moderate levels of error and fit. The comparative performance of the four models is illustrated in Figure 6, which visually displays the differences in predictive effectiveness among the models.

**FIGURE 7:**
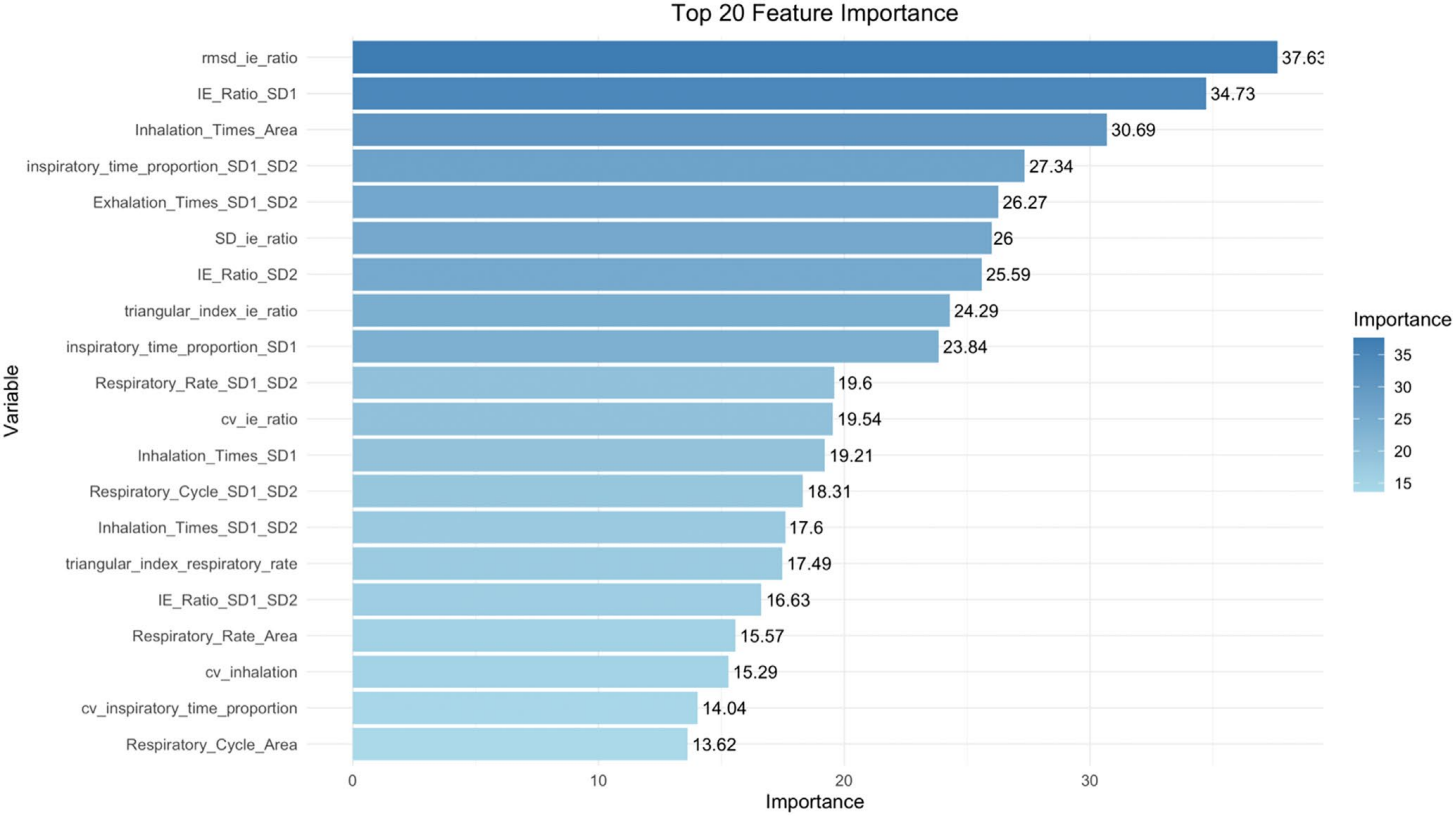
Top 20 important features selected by the Random Forest model and their importance rankings.

**FIGURE 8:**
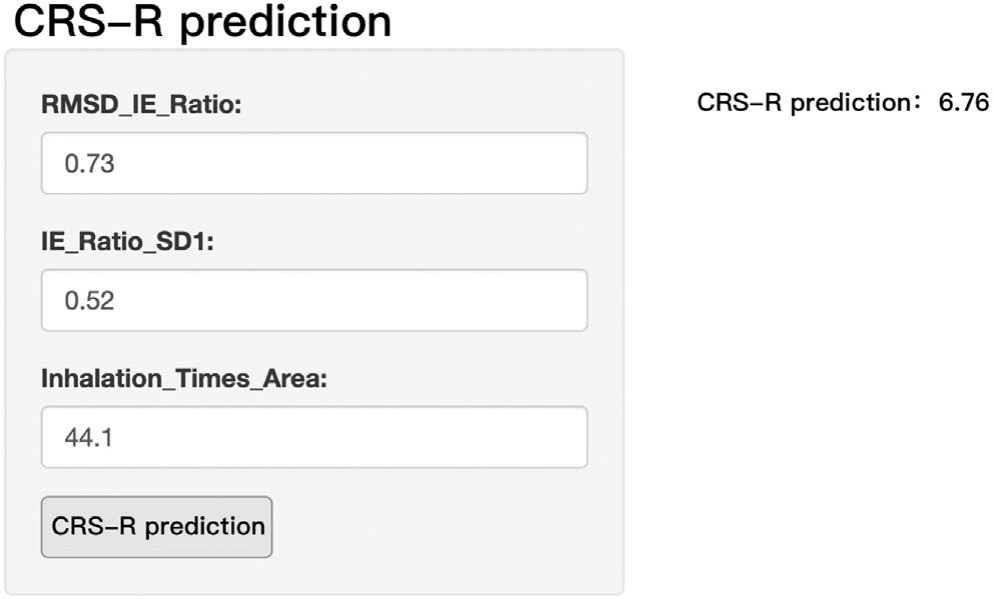
Interactive prediction interface based on the Random Forest model (the actual CRS‐R score for this patient is 6).

In addition, by performing variable importance analysis using the Random Forest model, we selected the most important feature variables and developed a preliminary interactive prediction interface (as shown in Figures 7 and 8). This interface allows users to input key RV indicators and predict the patient's CRS‐R score in real‐time, providing a more convenient tool for clinical application.

### 3.4 Construction of Clinical Outcome Prediction Model

**FIGURE 9:**
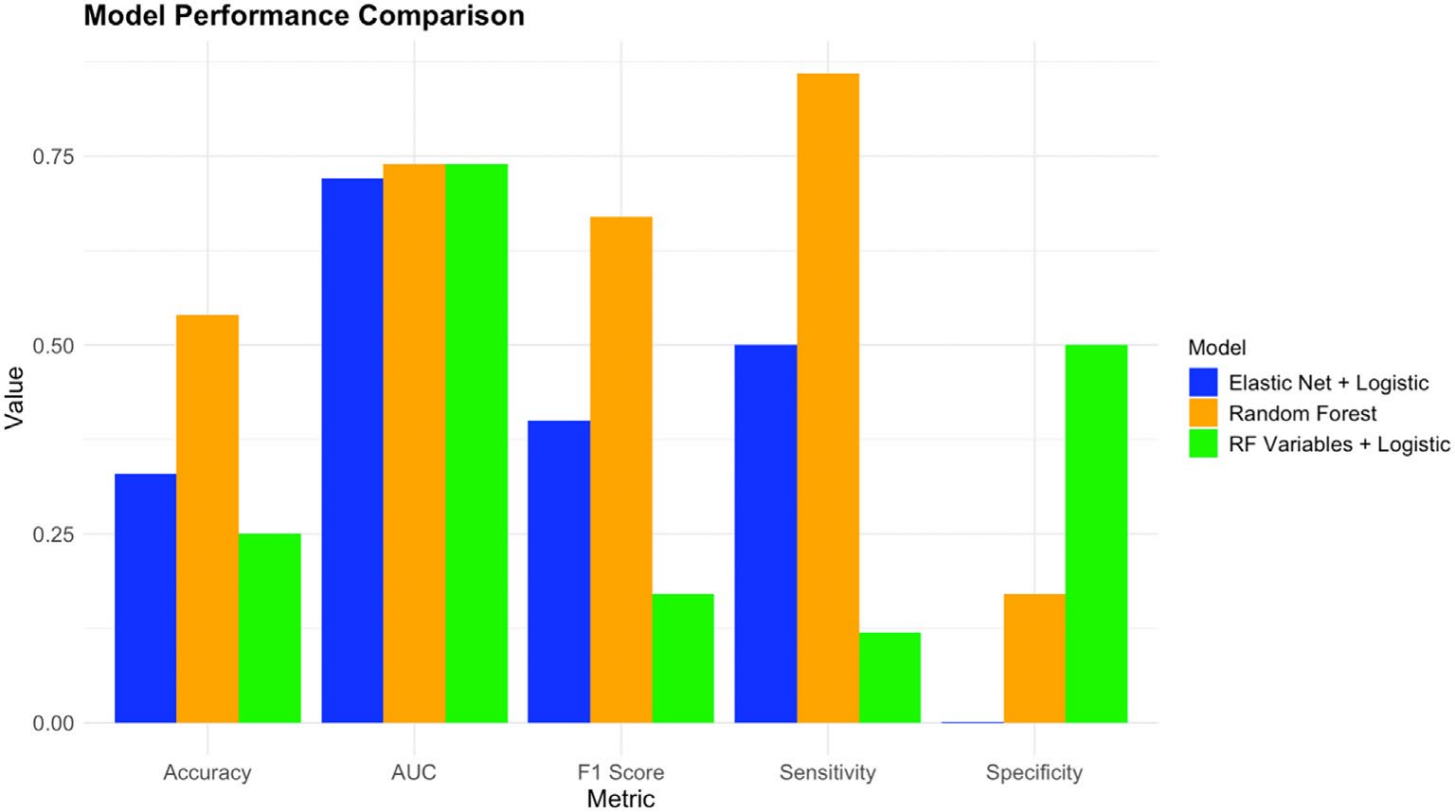
Comparison of performance metrics across different models. Accuracy, proportion of correct predictions; AUC, area under the ROC curve; F1 Score, harmonic mean of precision and recall; Sensitivity (Recall), true positive rate; Specificity, true negative rate.

In constructing the clinical outcome prediction model, we compared the performance of the Random Forest, Elastic Net+Logistic Regression, and Logistic Regression models based on Random Forest variable selection (see Figure 9). The results showed that the Random Forest model performed the best overall, with an AUC of 0.74, accuracy of 0.54, sensitivity as high as 0.86, and an F1 score of 0.67, indicating high accuracy and balance in predicting the “improvement” category of patients. In contrast, the Elastic Net+Logistic Regression and Logistic Regression models based on Random Forest variable selection had AUCs of 0.72 and 0.74, respectively, but lower accuracy rates of 0.33 and 0.25, with both sensitivity and F1 scores inferior to the Random Forest model. Overall, the Random Forest model demonstrated superior predictive ability in handling this type of binary classification task.

**FIGURE 10:**
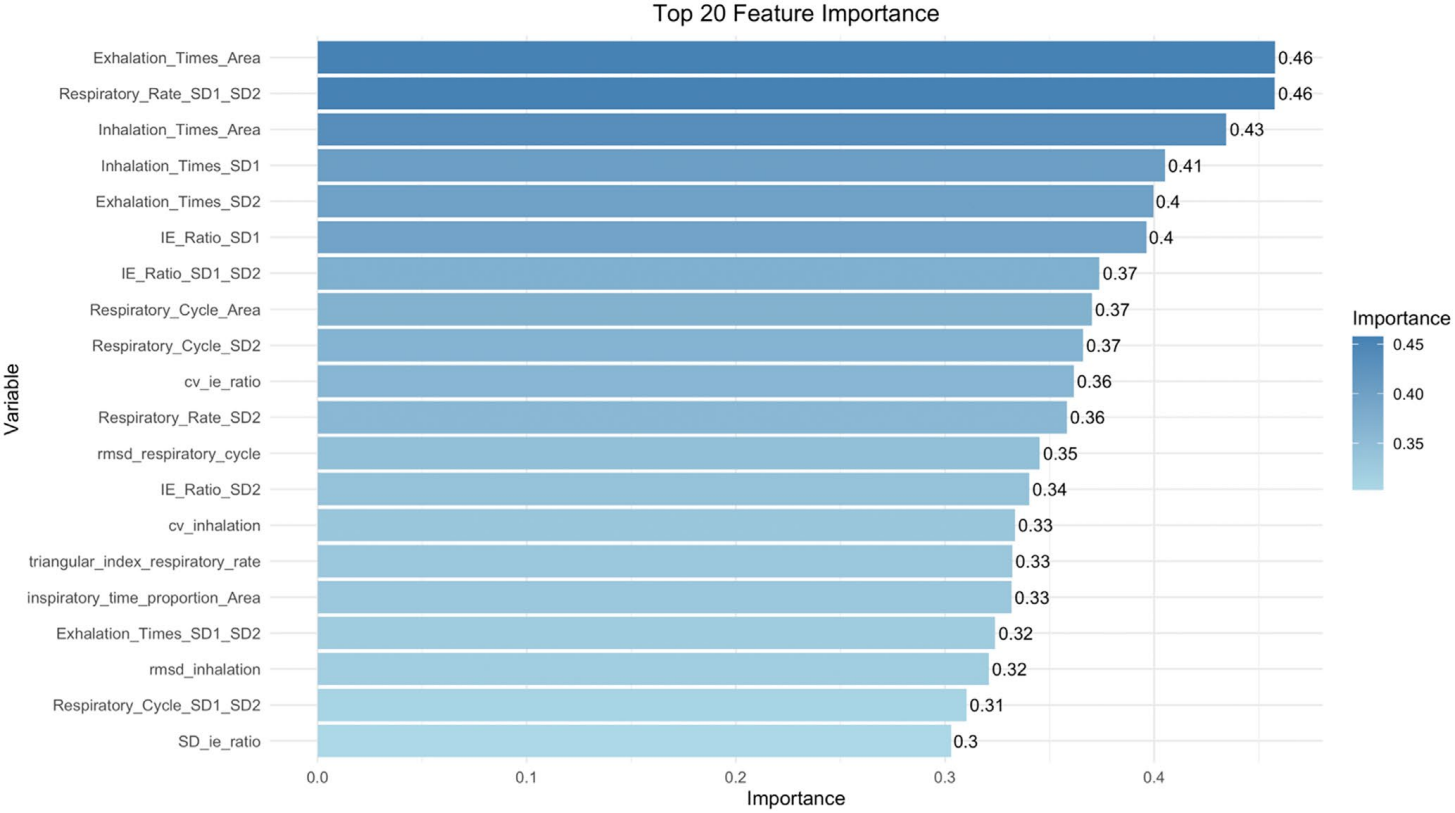
Variables selected by the Random Forest model.

Based on these evaluation results, we ultimately selected the Random Forest model as the optimal model and used the important features it identified (see Figure 10) to construct the clinical outcome prediction model. This model effectively predicts patient improvement and achieves high predictive accuracy on the validation set.

**FIGURE 11:**
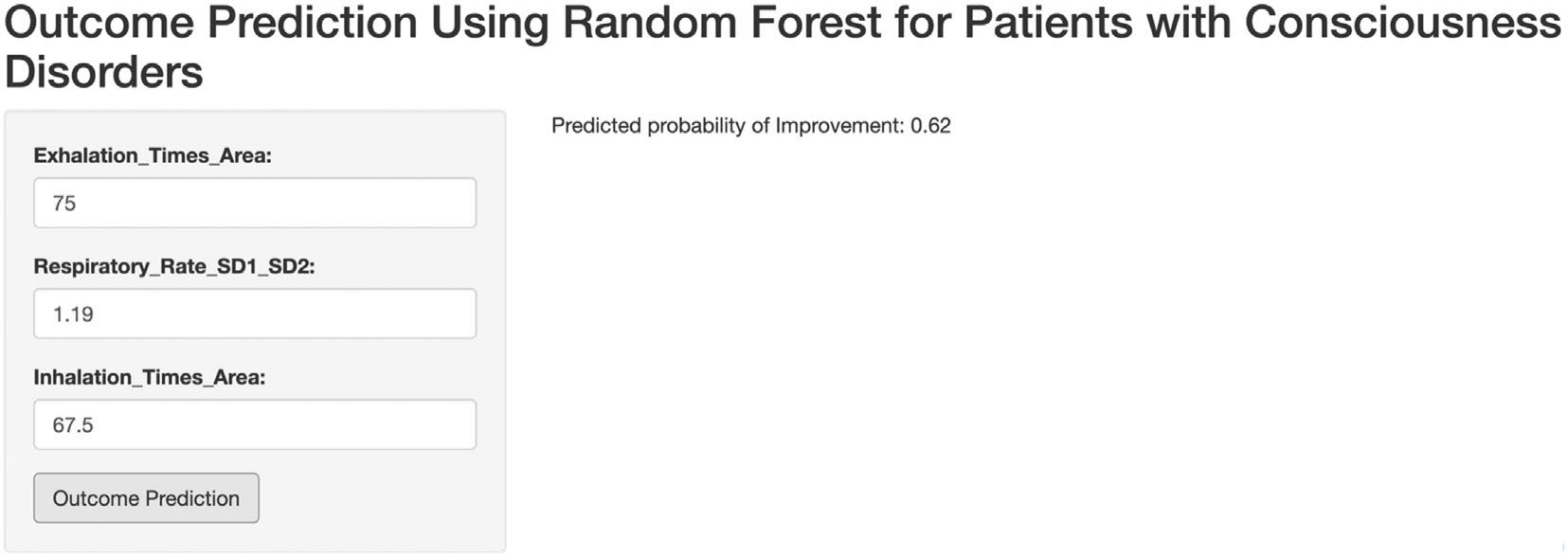
Clinical outcome prediction interface based on the Random Forest model.

To facilitate clinical application, we also developed an interactive prediction interface (see Figure 11). This interface allows clinicians to input key RV indicators of a patient and receive real‐time probability predictions for patient improvement. The interface is intuitive and easy to use, providing clinicians with real‐time decision support.

## 4 Discussion

This study explored the relationship between RV and levels of consciousness by thoroughly analyzing the differences in RV between patients with DOC and healthy controls. Using various statistical analysis methods and machine learning models, we found that RV has potential value in predicting the prognosis of patients with DOC. The results not only revealed significant changes in RV among these patients but also confirmed the superior performance of machine learning models, particularly the Random Forest model, in handling complex physiological data. In the following discussion, we will focus on three key aspects: the pathophysiological mechanisms of RV indicators, the robustness of statistical analyses, and the clinical application value of machine learning models.

### 4.1 Pathophysiological Mechanisms of RV and DOC

Currently, international guidelines recommend using a combination of clinical presentation, electroencephalography (EEG), electrocardiography (ECG), and neuroimaging to assess patients' levels of consciousness [21]. However, due to limitations in equipment and algorithms, the relationship between RV and consciousness levels has been rarely reported in previous literature.

The observed alterations in RV among patients with DOC provide compelling evidence of disrupted brainstem‐autonomic integration, offering new insights into the pathophysiology of impaired consciousness. Our findings demonstrate that DOC patients exhibit both increased nonlinear complexity (elevated inspiratory‐expiratory ratio SD2) and reduced overall respiratory flexibility (decreased triangular index), suggesting a dual pattern of dysregulation. The heightened complexity may reflect compensatory overactivity in residual brainstem circuits [22]—particularly the pre‐Bötzinger complex and ventral respiratory column—following damage to cortico‐brainstem pathways [23]. This is consistent with studies showing that DOC impairs top‐down modulation of autonomic function, leading to disinhibited, chaotic respiratory patterns [13]. Conversely, the loss of respiratory variability indicates a rigidification of autonomic control, likely due to sympathovagal imbalance and diminished physiological adaptability [24]. Such rigidity may underlie the blunted responsiveness to environmental stimuli characteristic of DOC, as the autonomic nervous system loses its capacity for dynamic adjustment [5].

These RV abnormalities align with well‐established biomarkers of autonomic dysfunction in DOC, including reduced heart rate variability and impaired pupillary reflexes [25], collectively underscoring the critical role of brainstem integrity in consciousness maintenance [26]. The significant correlations observed between specific RV parameters (e.g., SD1) and standardized clinical assessments (e.g., CRS‐R scores) demonstrate that respiratory patterns may serve as a practical, bedside‐compatible biomarker for evaluating brainstem functional integrity. This clinical utility is particularly valuable considering the technical challenges of obtaining neuroimaging in unresponsive patients.

Notably, the predictive value of RV parameters parallels that of other brainstem functional assessments, such as auditory brainstem responses (ABRs) and somatosensory evoked potentials (SEPs) [27, 28], which have been widely used to evaluate pathway integrity and predict clinical outcomes in DOC patients. Just as these electrophysiological markers reflect the functional status of specific neural pathways, RV parameters provide a comprehensive assessment of the respiratory control network, encompassing both brainstem nuclei and their cortical connections.

The convergence of our findings with prior work on physiological complexity measures—including Piergiuseppe et al.'s EEG‐based complexity indices [29] lends further support to the emerging paradigm that consciousness emerges from integrated, multiscale neural dynamics. Importantly, the non‐invasive nature and continuous monitoring capability of RV measurement offer distinct advantages over intermittent electrophysiological testing, positioning it as a promising complementary tool for both clinical assessment and neurobiological investigation of consciousness disorders.

Moving forward, research should prioritize longitudinal studies to determine whether RV patterns predict recovery trajectories or treatment responses. Additionally, multimodal approaches combining RV with fMRI or PET imaging could clarify the specific neural substrates of respiratory dysregulation in DOC. Future studies exploring the combined use of RV parameters with traditional evoked potential testing may yield more robust prognostic models for DOC patients. The noninvasive nature of RV measurement positions it as a promising tool for both mechanistic research and clinical monitoring, potentially bridging the gap between bedside assessment and neurobiological understanding of consciousness disorders.

### 4.2 Application of Statistical Methods and Robustness Analysis

Previous studies have used sensors for detecting respiratory rate. Torres et al. were the first to demonstrate the feasibility of using a uniaxial accelerometer to monitor respiratory signals in a canine model, using a tracheal pressure sensor as a reference [30]. The IMU sensor used in this study supports the detection of triaxial acceleration signals, providing a solid foundation for selecting stable signals [31]. In our preliminary work, we also validated the high accuracy of this sensor in capturing respiratory signals in a healthy population.

To ensure the robustness of our analysis, we used a GAM to adjust for various confounding factors, including age, gender, body temperature, heart rate, and pulse pressure difference. In particular, the smoothing function s(age) was employed to capture potential nonlinear relationships between age and RV. The introduction of GAM allowed us to more accurately adjust for the influence of confounding factors on RV, ensuring the rigor of the analysis. Moreover, the application of the Bootstrap resampling method further enhanced the robustness of the results. Through 1000 resampling iterations, we calculated 95% confidence intervals for each index, compensating for the limitations of a small sample size.

Additionally, to address issues of asymmetric data distribution and small sample size, we used the Mann–Whitney *U* test as a substitute for the traditional *t*‐test. This non‐parametric test avoids reliance on the assumption of normal data distribution, making it more suitable for the characteristics of our data. Lastly, to control for the risk of false positives due to multiple comparisons, we introduced FDR correction, ensuring the statistical reliability of all significant results.

### 4.3 Comparison and Application Value of Machine Learning Models

Our comparative analysis revealed that the Random Forest (RF) model demonstrated superior performance in analyzing multidimensional respiratory variability (RV) data, outperforming Elastic Net, PLS, and SVM models in predicting both CRS‐R scores (with lower MSE/RMSE and higher *R* ^2^) and clinical outcomes (with higher AUC, accuracy, and F1 scores). The RF model's ability to capture complex nonlinear relationships in physiological data proved particularly valuable for DOC prognosis assessment. Recognizing the critical importance of sensitivity in clinical decision‐making for DOC patients—where missing potential improvements (false negatives) carries greater consequences than false alarms. This approach aligns with clinical priorities for early detection of improvement opportunities to guide therapeutic decisions.

While these results highlight the potential of RV as an autonomic nervous system biomarker to complement standard CRS‐R assessments [32], we emphasize the need for future work to: (1) develop hybrid approaches (e.g., ensemble methods) to better balance sensitivity/specificity, (2) validate findings in larger multicenter cohorts, and (3) integrate RV with other physiological markers to enhance prognostic accuracy. These advancements may ultimately support more personalized management strategies for DOC patients.

### 4.4 Study Limitations and Future Directions

While our study employed rigorous methods including bootstrap resampling and FDR‐corrected non‐parametric testing, several limitations warrant consideration:

First, the sample size of 44 patients, though carefully analyzed, remains below the ideal threshold (*n* = 110/group) for FDR‐corrected analyses, which may affect the precision of our effect size estimates. Second, the cross‐sectional design prevents causal interpretation of the observed RV‐consciousness relationships. Third, while signal quality was validated in healthy volunteers, extreme abdominal adiposity in some patient populations could theoretically affect sensor performance—though this concern is mitigated by standardized body positioning and our adaptive drift‐correction algorithms. Finally, although we controlled for major physiological confounders using GAMs, potential influences from unmeasured respiratory complications cannot be entirely ruled out [33]. It is particularly important to note that the current analysis of respiratory variability is limited to time‐domain analysis, failing to comprehensively capture the multidimensional characteristics of respiratory signals.

Future research should first focus on addressing the clinical application of the model by further optimizing algorithms and interfaces, enabling it to conveniently assess and monitor patients' levels of consciousness in real‐world medical settings, thereby directly ensuring adequate sample sizes and supporting clinical decision‐making. Secondly, there is a need for long‐term dynamic monitoring that integrates more physiological and neurological data to capture trends and potential risks in patients' conditions. This requires multidisciplinary collaboration to achieve comprehensive data integration and analysis. Lastly, by incorporating artificial intelligence, deep learning algorithms can be used to enhance the accuracy of analyzing RV and other physiological signals, enabling more precise prognosis predictions and exploring the underlying pathophysiological mechanisms of RV in disorders of consciousness. Notably, future studies should expand the analytical dimensions by incorporating methods such as frequency‐domain analysis, fractal analysis, and entropy analysis into the research framework. This multidimensional approach will enable a comprehensive characterization of respiratory variability and a deeper exploration of its association with DoC. Advancements in these research directions are expected to provide new theoretical foundations and technical support for the precise assessment and personalized treatment of patients with DoC.

Although we focused on characterizing intrinsic RV patterns—a necessary first step for biomarker development—our findings also scaffold targeted intervention studies. Specifically, the identified RV abnormalities (e.g., elevated inspiratory‐time‐proportion) may serve as physiological readouts for future neuromodulation trials. Beyond targeted interventions, our RV framework could synergize with emerging multi‐omics approaches, such as integrating multi‐omics data with RV biomarkers for comprehensive patient stratification [34].

## 5 Conclusion

This study highlights the close relationship between RV and DOC. By applying machine learning models, particularly the Random Forest model, we demonstrated the potential application of RV in prognostic assessment for patients with DOC. These findings not only deepen our understanding of the physiological mechanisms underlying DOC but also provide new perspectives and guidance for clinical treatment and monitoring.

## Funding

This work was supported by the Suzhou Multi‐Center Clinical Research Project for Major Diseases (Grant No. DZXYJ202524) and the Key Research and Development Plan of Jiangsu Province (Social Development) Project (Grant No. BE2021618).

## Conflicts of Interest

The authors declare no conflicts of interest.

## Supporting information


**Appendix S1:** cns70812‐sup‐0001‐Appendix.docx.

## Data Availability

The data that support the findings of this study are available from the corresponding author upon reasonable request.
