# Supplementary material for: Respiratory Variability in Disorders of Consciousness: Relationship and Clinical Applications
Source: CNS Neurosci Ther. 2026 May 12;32(5):e70812. doi: 10.1002/cns.70812 (PMC13163149; doi:10.1002/cns.70812)
Supplement: Supplementary file 1 — Appendix S1: cns70812‐sup‐0001‐Appendix.docx. [file CNS-32-e70812-s001.docx]

Appendix

We evaluated the use of inertial measurement unit (IMU) sensors placed on the abdomen in a supine position to measure Ti and Te. Twelve healthy volunteers participated, and respiratory signals were collected over 5 minutes. Using a multi-axis measurement approach, acceleration data were processed to reconstruct respiratory curves and extract Ti and Te values. Results from 954 respiratory cycles showed mean Ti and Te values of 1.78±0.21s and 1.83±0.25s,with absolute errors of 0.10±0.04s and 0.11±0.05s, and relative errors of 5.95%±2.8% and 7.21%±5.6%. High correlation coefficients (0.81 for Ti, 0.83 for Te) and Bland-Altman analysis confirmed strong measurement accuracy and reliability. The IMU sensor method provides a non-invasive, portable solution for clinical and home respiratory monitoring, offering significant clinical application potential.

Based on the normal human breathing frequency range (0.2-0.34 Hz) and Nyquist theorem, to avoid signal aliasing, the sensor's sampling frequency was set at 10 Hz. After data collection, the raw acceleration data was processed using R language. To effectively extract respiratory signals, a Butterworth filter and moving average method were applied. Specifically, the filter's low and high cutoff frequencies were set at 0.1 Hz and 1 Hz to remove unwanted noise, and a second-order filter along with a moving average window of length 16 was used to smooth the signal and reduce high-frequency noise.

During the analysis, the first step was to calculate the standard deviation of the intervals between peaks and troughs for each acceleration component. The axis with the smallest overall standard deviation was selected as the reference axis, and the second smallest as the target axis. After identifying the reference and target axes, the waveforms were standardized based on the same baseline and coordinate system. For respiratory signals, inspiration was determined by whether the waveform was rising or falling. Depending on the direction, inspiration and expiration times were calculated accordingly (see Figure 1).


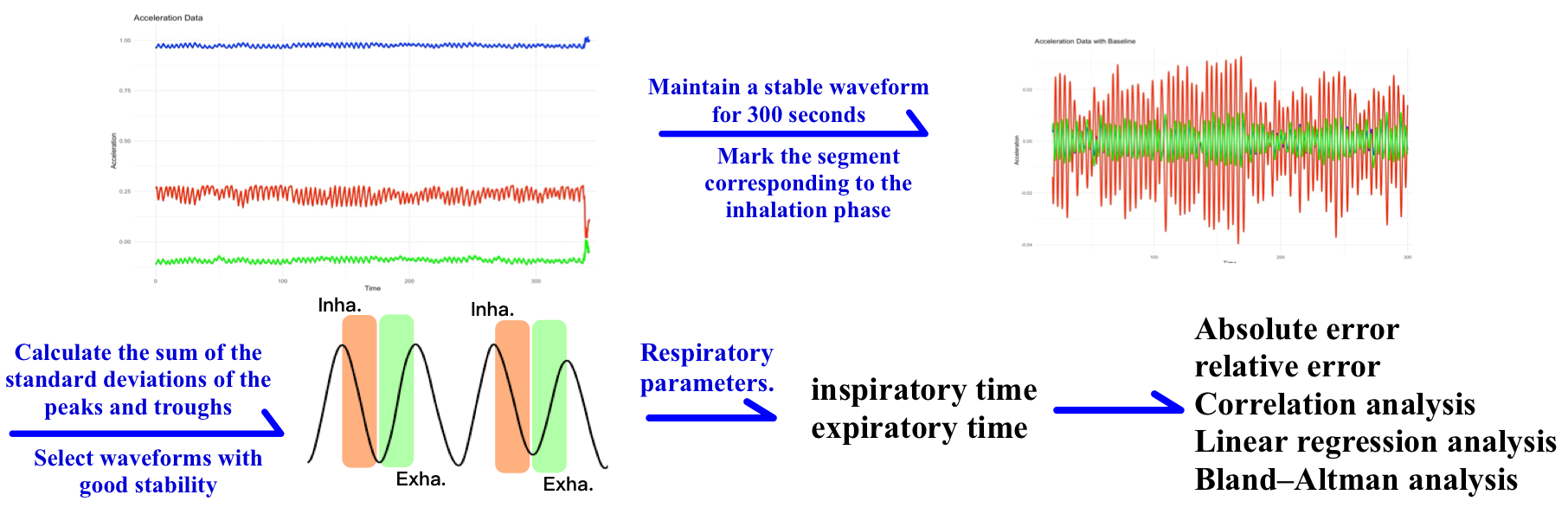


**Figure 1:** the process of extracting and processing respiratory signals

Twelve healthy volunteers were recruited, and all experiments were conducted under the same environmental conditions. Volunteers lay supine on a hard bed, with the sensor secured between the xiphoid process and the navel using tape. Participants were instructed to breathe naturally and steadily throughout the experiment to ensure accurate and consistent data collection. The basic information of volunteers is shown in Table 1.

**Table 1:** The basic information of volunteers

| **Participant** | **Gender** | **Age (years)** | **Height (cm)** | **BMI(kg·m^-2^)** |  |
| --- | --- | --- | --- | --- | --- |
| **1** | Male | 42 | 173 | 23.39 |  |
| **2** | Male | 22 | 182 | 22.94 |  |
| **3** | Male | 25 | 170 | 20.76 |  |
| **4** | Male | 25 | 173 | 26.73 |  |
| **5** | Male | 24 | 175 | 26.12 |  |
| **6** | Male | 24 | 180 | 28.09 |  |
| **7** | Female | 50 | 155 | 20.81 |  |
| **8** | Female | 59 | 157 | 23.94 |  |
| **9** | Female | 22 | 172 | 30.42 |  |
| **10** | Female | 22 | 162 | 18.67 |  |
| **11** | Female | 24 | 175 | 19.59 |  |
| **12** | Female | 49 | 158 | 21.63 |  |

As shown in Figure 2, data from the reference axis for 954 respiratory cycles across 12 subjects indicate an average inspiratory time of 1.78±0.21 seconds and an average expiratory time of 1.83±0.25 seconds. According to Figure 3, the 95% confidence intervals for the mean errors between the inspiratory and expiratory times on the target and reference axes both include zero, suggesting that there are no statistically significant differences in respiratory parameters between the two axes.

**
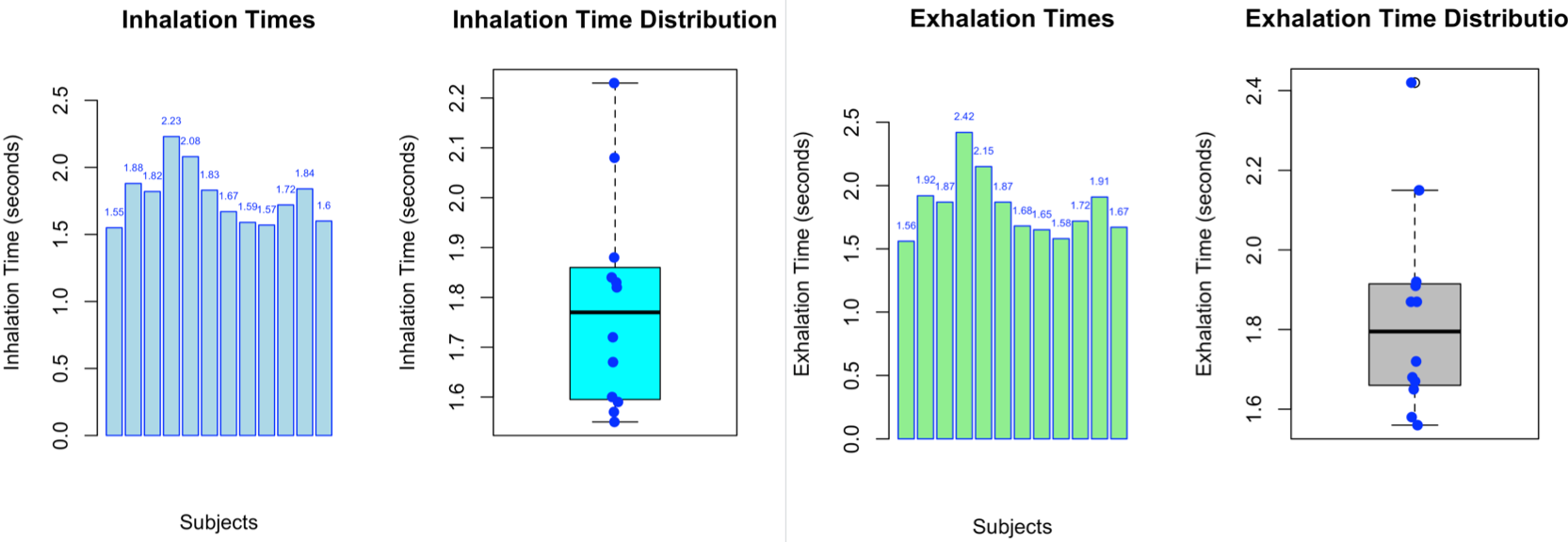
**

**Figure 2:** Distribution of inspiratory and expiratory times on the reference axis


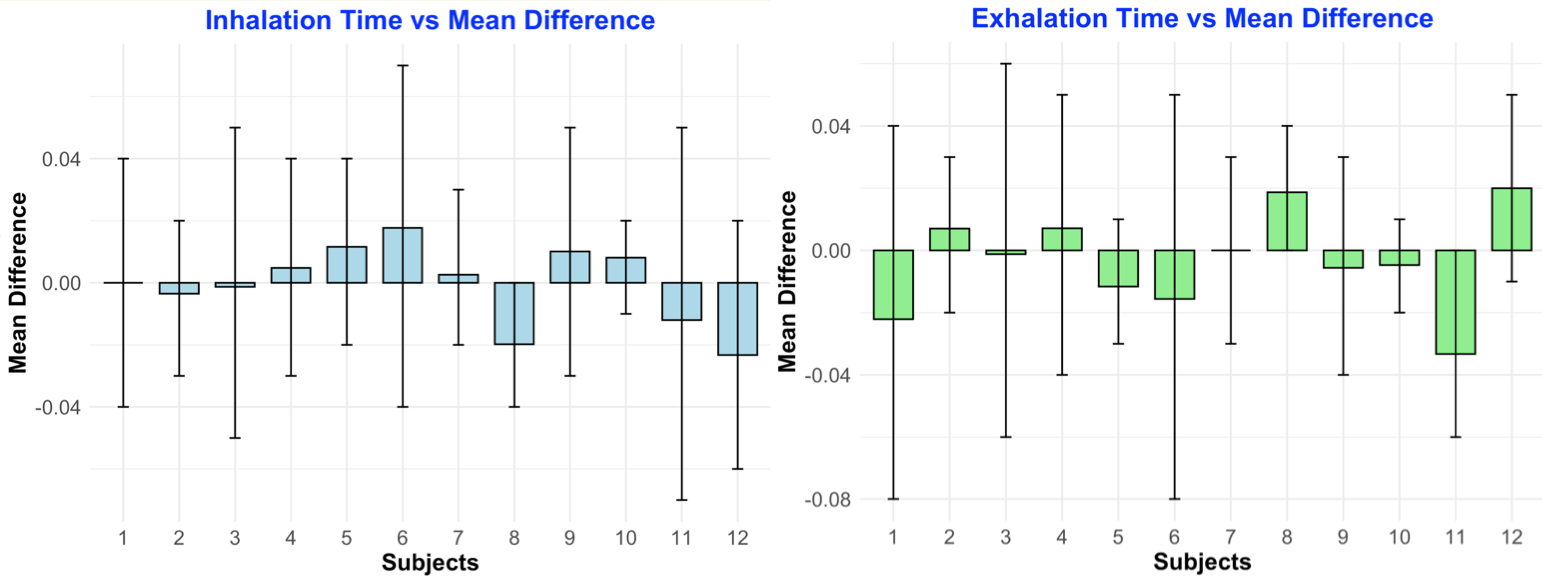


**Figure 3:** Error analysis of inspiratory and expiratory times between the reference and target axes

Figure 4 presents box plots showing the absolute and relative errors of inspiratory and expiratory times across 12 subjects for both axes. The results indicate that the absolute errors for inspiratory and expiratory times are E = 0.10 ± 0.04 and E = 0.11 ± 0.05, respectively. The relative errors are E % = 5.95% ± 2.8% for inspiratory time and E % = 7.21% ± 5.6% for expiratory time. These results demonstrate that the sensor exhibits high accuracy and consistency in measuring both inspiratory and expiratory times.


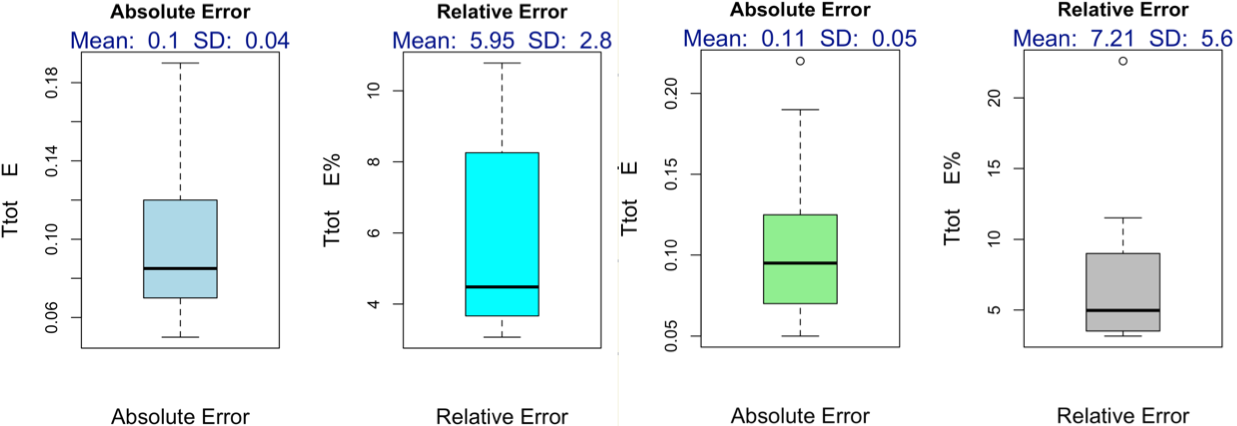


**Figure 4:** Box plots of absolute and relative errors for inspiratory and expiratory times

Figures 5 and 6 show scatter plots of inspiratory and expiratory times from 12 subjects. Linear regression analysis reveals a significant linear relationship between the respiratory cycles on the two axes. The correlation coefficients for inspiratory time and expiratory time are 0.81 and 0.83, respectively, indicating a high level of consistency in respiratory parameters captured on both axes.


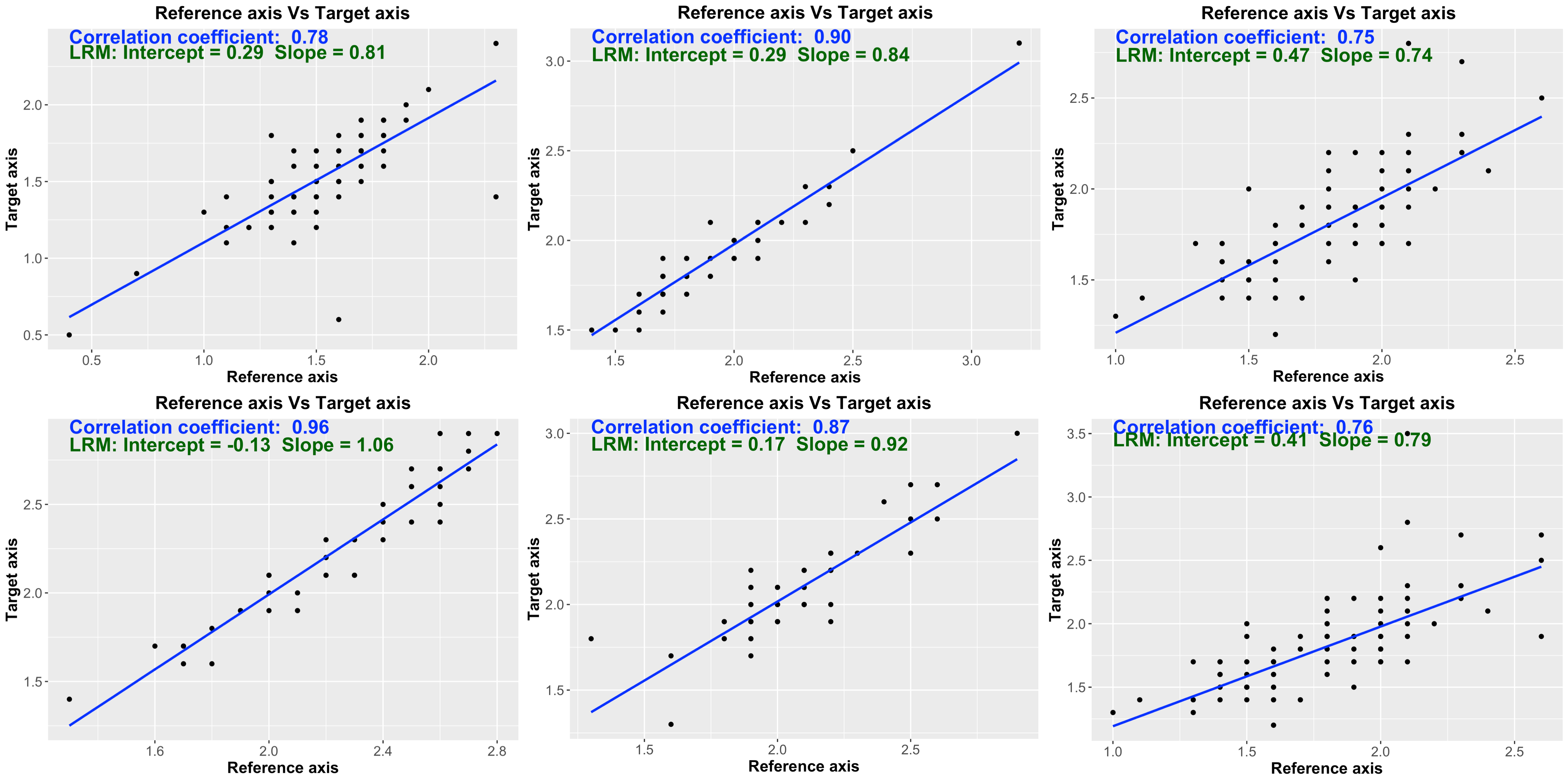


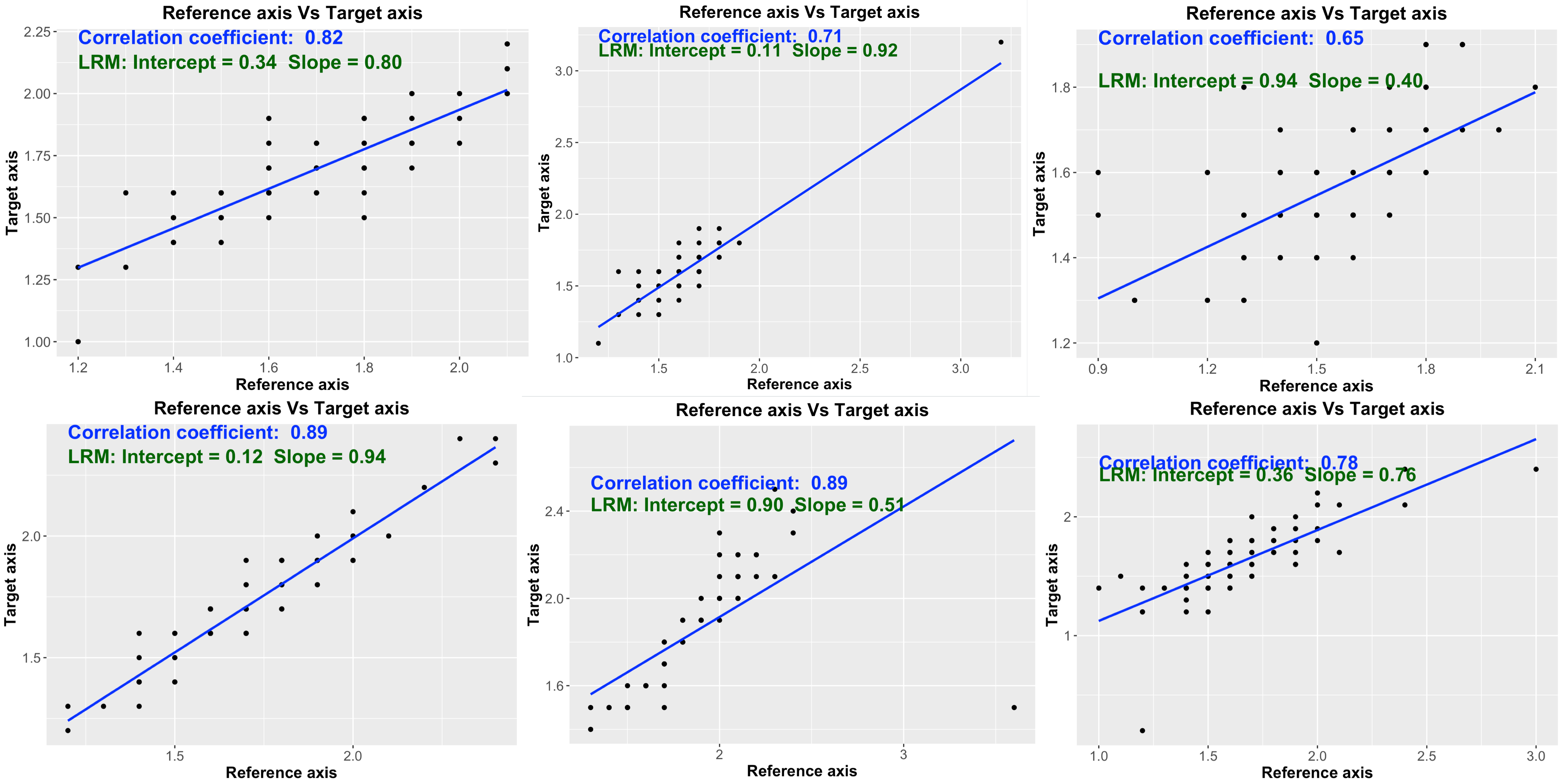


**Figure 5:** Linear regression and correlation analysis of inspiratory times for 12 subjects


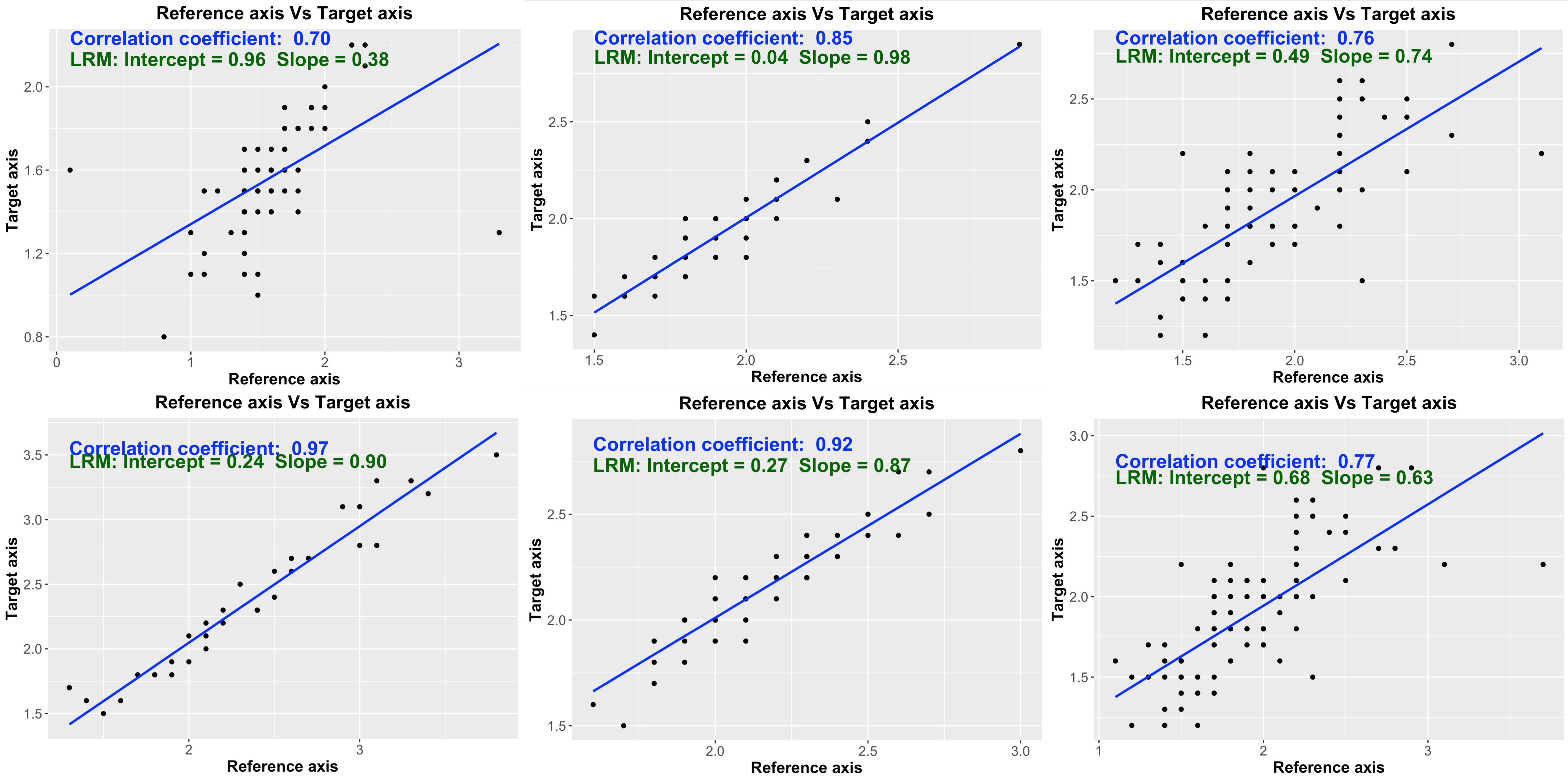


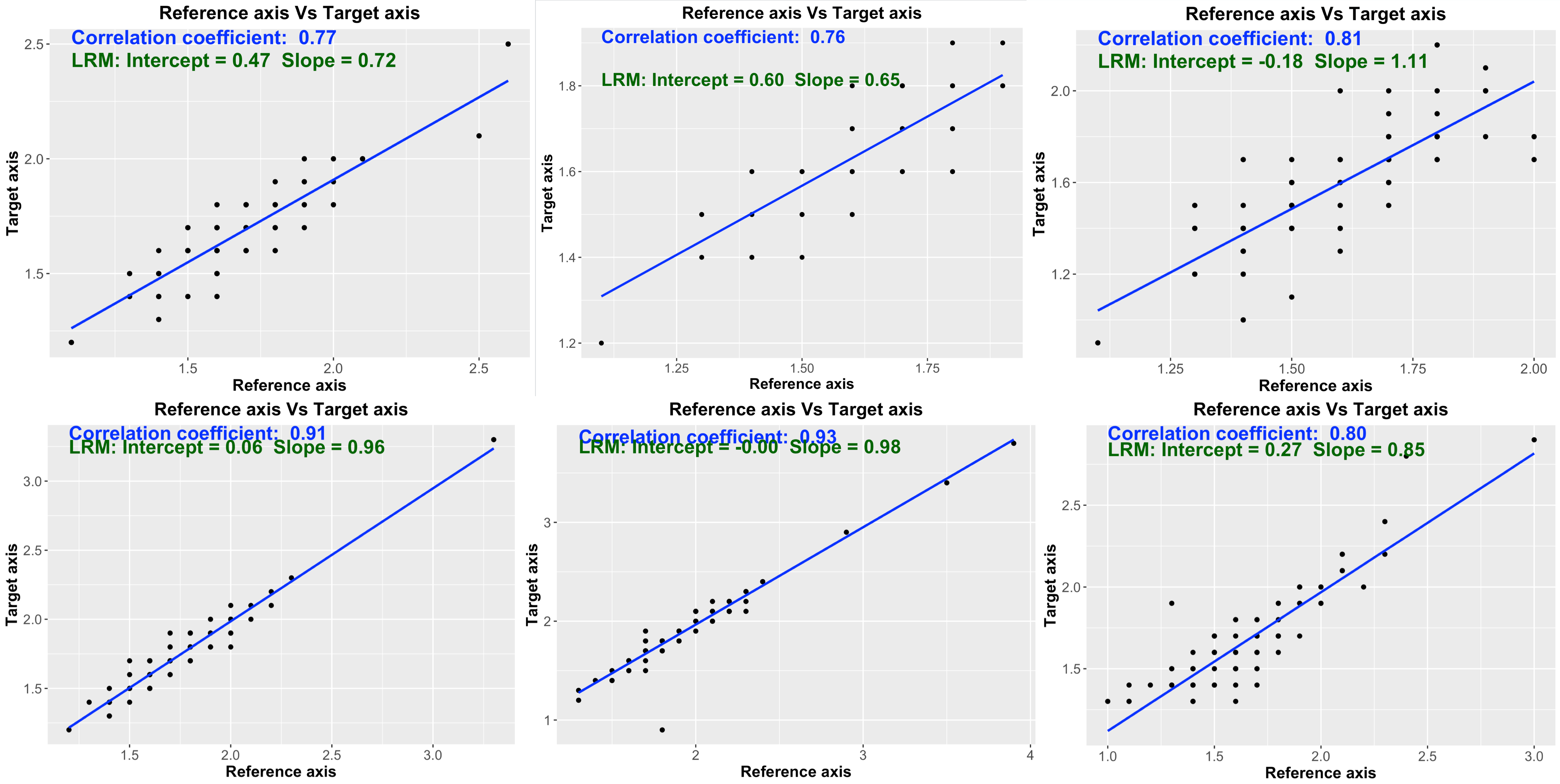


**Figure 6:** Linear regression and correlation analysis of expiratory times for 12 subjects

Figures 7 and 8 demonstrate the good agreement of the sensor in monitoring inspiratory and expiratory times using Bland-Altman plots. These plots depict the differences between the target and reference axes measurements relative to their means, assessing bias and consistency. The red dashed line represents the average difference, while the blue dashed lines indicate the limits of agreement (±1.96 seconds). Most data points fall within this range, indicating high consistency. Additionally, an OLS regression line (green) shows any trend in the differences, confirming the sensor's accuracy and reliability in capturing respiratory times.

**
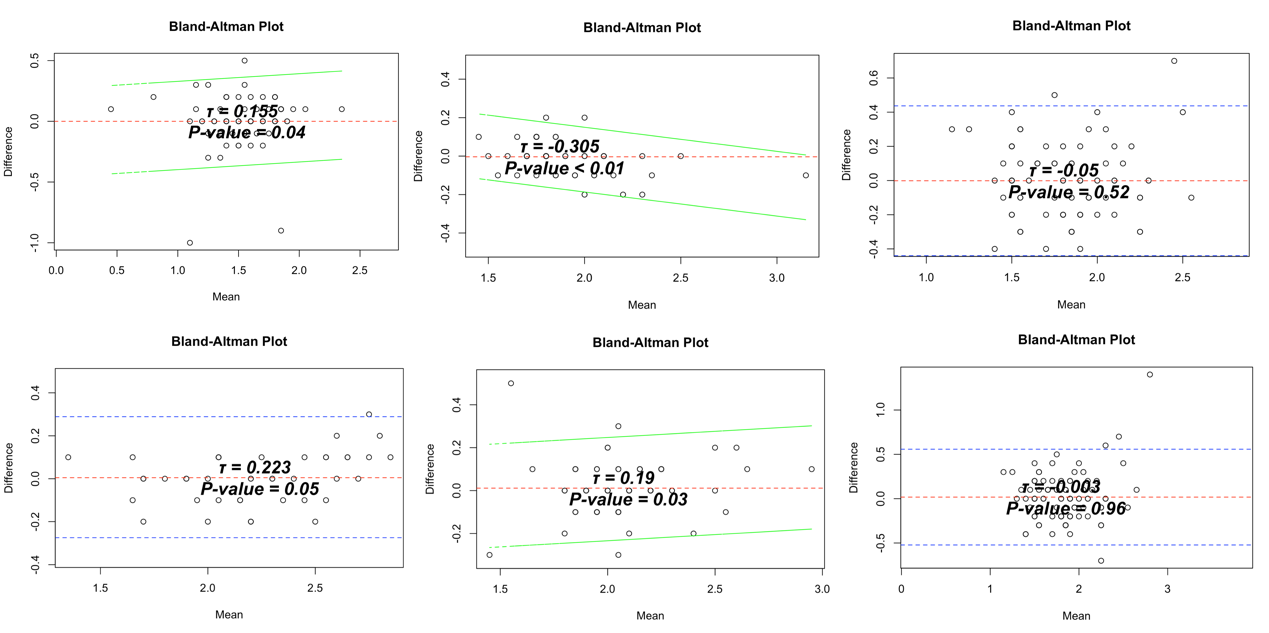
**

**
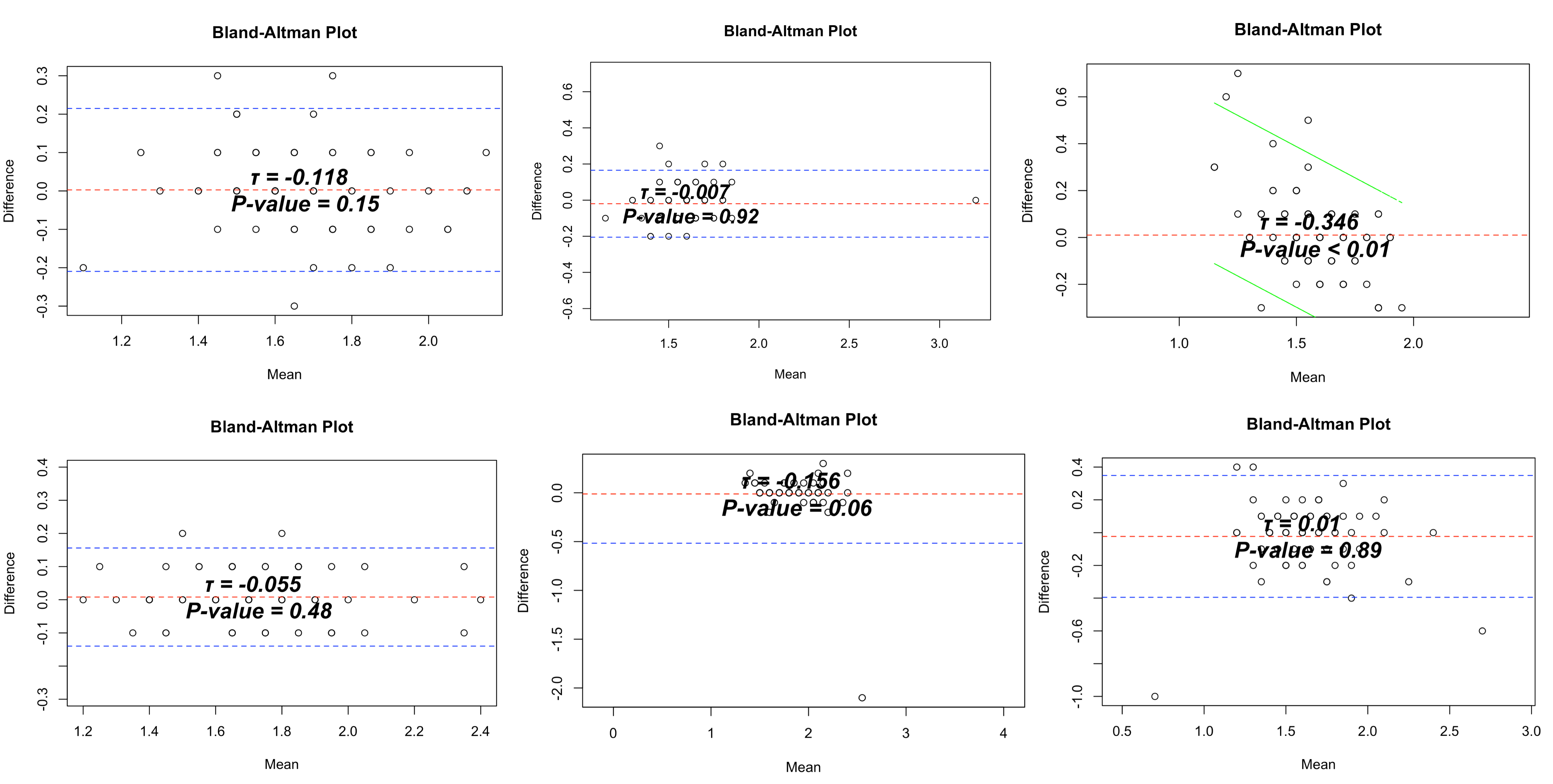
**

**Figure 7:** Consistency analysis of inspiratory time between the target and reference axes


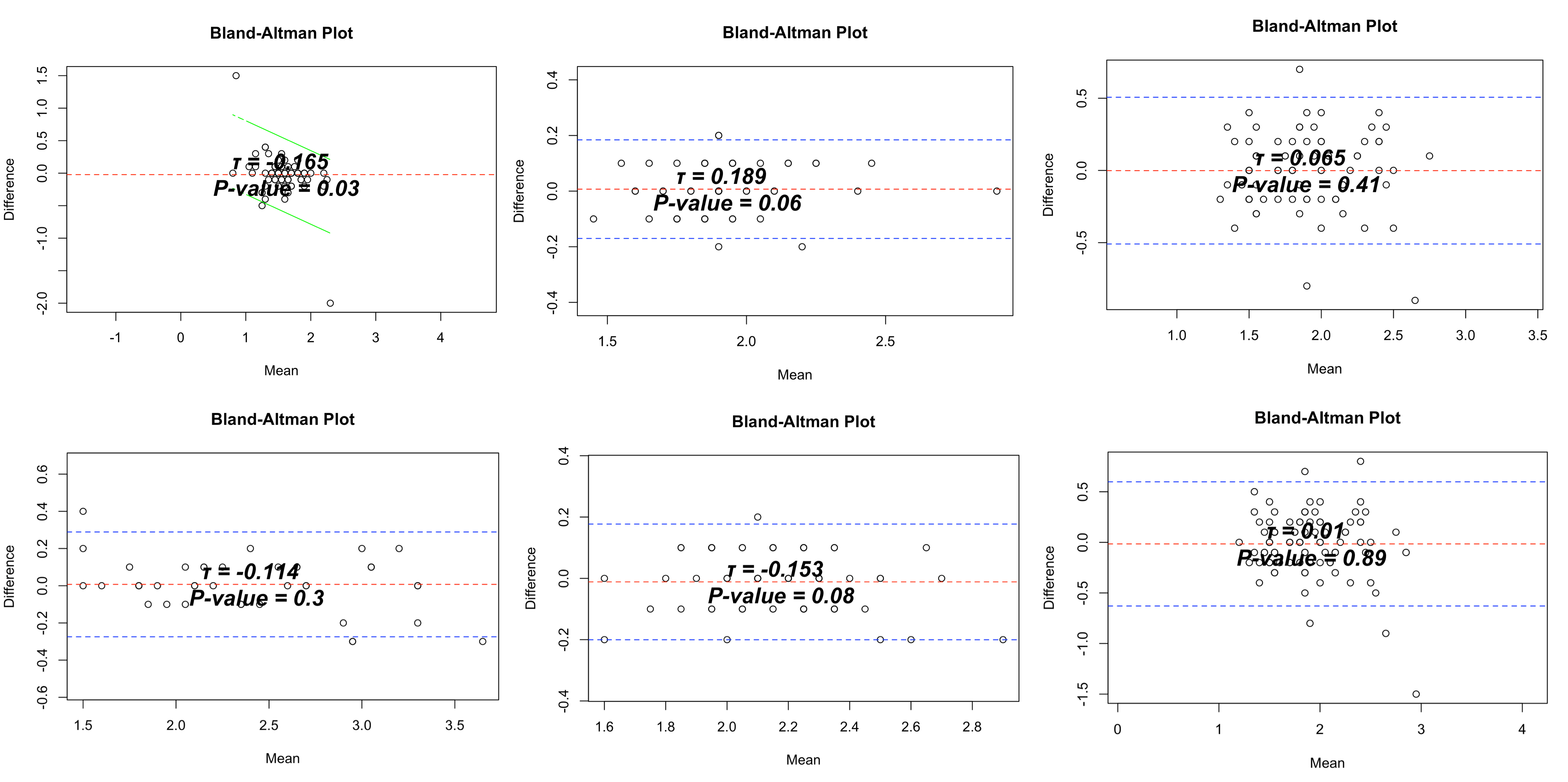


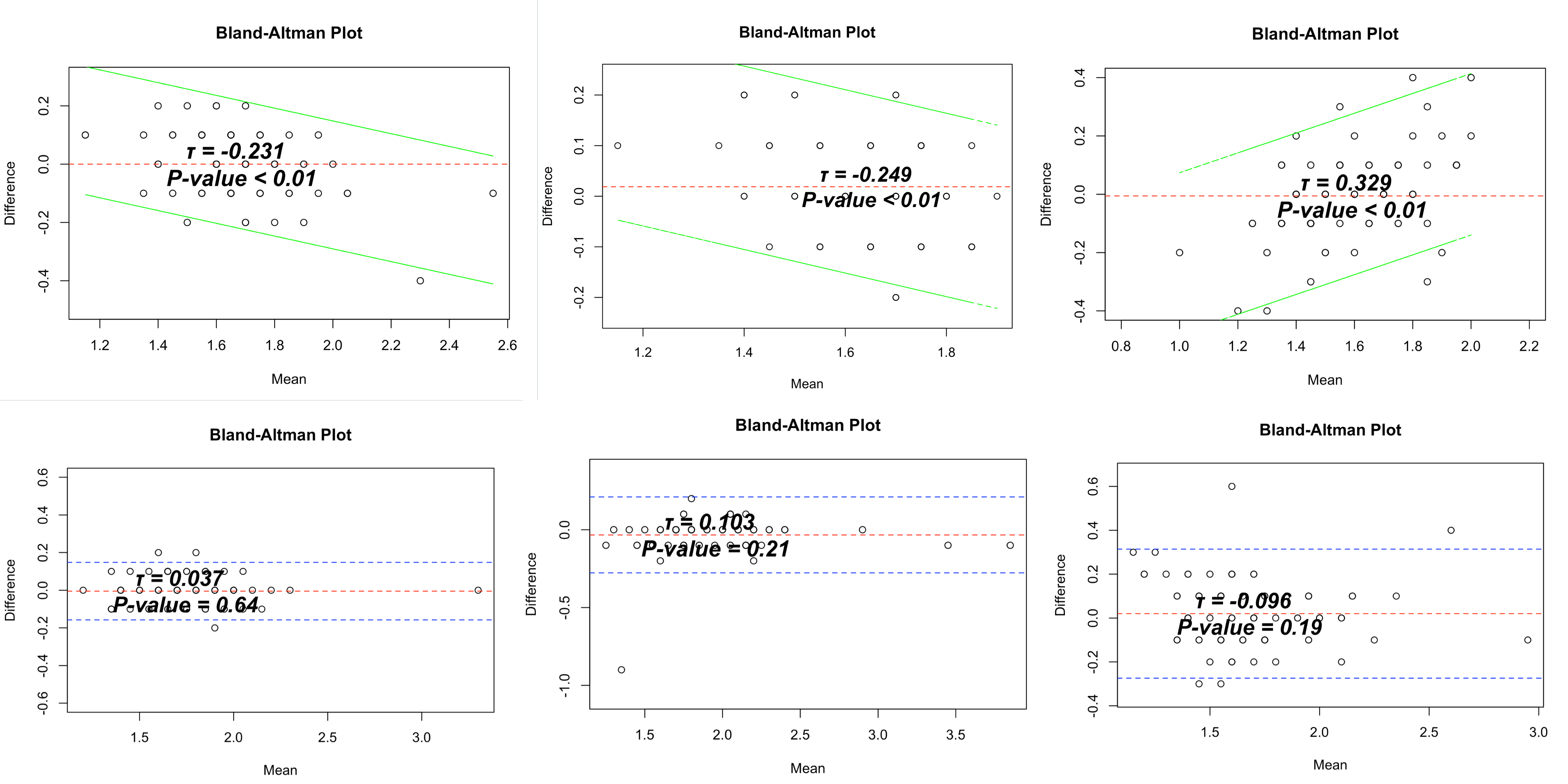


**Figure 8:** Consistency analysis of expiratory time between the target and reference axes

In conclusion, this study has validated the high accuracy and consistency of IMU sensors in measuring Ti and Te , providing a more comprehensive and detailed assessment tool for clinical respiratory monitoring. By exploring the clinical significance of Ti and Te, the sensors show great potential in the diagnosis, treatment monitoring, and rehabilitation training for respiratory diseases. With further technological advancements and broader application, IMU sensors could offer new opportunities in respiratory health, improving patient care and quality of life.
